# Rationale and development of a survey tool for describing and auditing the composition of, and flows between, specialist and community clinical services for sexually transmitted infections

**DOI:** 10.1186/1472-6963-11-30

**Published:** 2011-02-09

**Authors:** Catherine RH Aicken, Jackie A Cassell, Claudia S Estcourt, Frances Keane, Gary Brook, Greta Rait, Peter J White, Catherine H Mercer

**Affiliations:** 1 Centre for Sexual Health and HIV Research, Research Department of Infection and Population Health, University College London, Mortimer Market Centre, off Capper Street, London WC1E 6JB, UK; 2 Brighton and Sussex Medical School, Mayfield House, University of Sussex, Falmer, Brighton BN1 9PH, UK; 3 Centre for Infectious Disease: Sexual Health and HIV Institute of Cell and Molecular Science, Barts and the London School of Medicine and Dentistry, Barts Sexual Health Centre, St Bartholomew's Hospital, London EC1A 7BE, UK; 4 Infection and Immunity, Barts and the London NHS Trust, London UK; 5 Department of Genito-urinary Medicine, Royal Cornwall Hospitals NHS Trust, The Hub, Royal Cornwall Hospital (Treliske), Truro, Cornwall TR1 3LJ, UK; 6 Patrick Clements Clinic, Central Middlesex Hospital, North West London Hospitals NHS Trust, Acton Lane, London NW10 7NS, UK; 7 Research Department of Primary Care and Population Health, University College London Medical School, Royal Free Campus, Rowland Hill Street, London NW3 2PF, UK; 8 MRC Centre for Outbreak Analysis & Modelling, Department of Infectious Disease Epidemiology, Imperial College Faculty of Medicine, London W2 1PG, UK; 9 Modelling & Economics Unit, Health Protection Agency, 61 Colindale Avenue, London NW9 5EQ, UK

## Abstract

**Background:**

National health strategies have called for an expansion of the role of primary care in England to increase access to sexual health services. However, there is little guidance for service planners and commissioners as to the public health impact of different combinations of specialist genitourinary medicine (GUM) clinics and primary care based services for local populations. Service planning for infectious diseases like sexually transmitted infections (STI) is further complicated because the goal of early detection and treatment is not only to improve the health of the individual, but to benefit the wider population and reduce future treatment costs by preventing onward transmission. Therefore, we are developing a survey tool that will enable service planners to better understand the needs of their local STI care-seeking population and which will help inform evidence-based decision-making about current and future service configurations. Here we describe the rationale and development of this survey tool.

**Methods/Design:**

A pen-and-paper questionnaire asking about sociodemographics, reasons for attendance, care pathways, and recent sexual risk behaviours, is being developed for patients to complete in waiting rooms of diverse clinical services, including GUM clinics and primary-care based services in sociodemographically- and geographically-contrasting populations in England. The questionnaire was cognitively tested before being piloted. In the pilot, 67% of patients participated, of whom 84% consented to our linking their questionnaire to data on STI testing and diagnosis and partner notification outcomes from their clinical records.

**Discussion:**

The pilot study suggests that both the questionnaire and its linkage to routinely-collected clinical data are likely to be acceptable to patients. By supplementing existing surveillance, data gathered by the survey tool will inform service planners' and providers' understanding of the needs and care-pathways of their patients, facilitating improved services and greater public health benefit.

## Background

Testing and treatment for sexually transmitted infections (STIs) in England has historically largely taken place in genitourinary medicine (GUM) clinics, although an increasing amount is occurring in primary care.[1] This reflects the recommendations of the National Strategy for Sexual Health and HIV[2], and more recently, the MedFASH/BASHH *Standards for the Management of Sexually Transmitted Infections (STIs)*[3] call to expand the role of primary care further to increase access to sexual health services. As a result, there has been growth in more specialised but highly variable[4,5] models of Local Enhanced Services for Sexual Health (LESSH) in primary care.[6] However, the published recommendations fail to provide commissioners and planners of sexual health services guidance as to how to decide on the relative capacity and characteristics of these clinical services to meet the sexual health needs of local populations, making local commissioning difficult.[4,5] Service planning for infectious diseases like STIs is further complicated because each case can produce further cases,[7] so the goal of early detection and treatment is not only to improve the health of the individual but that of the wider population by preventing onward transmission, which also reduces future treatment costs.[8] Cost-efficient services therefore need to provide rapid and appropriate care, tailored to the needs of their local populations.

The MSTIC study, an abbreviation of 'Maximising STI Control' (full study title: *'Public health outcomes of GUM and primary care-based STI services: How to maximise STI control for a population'*), is a UK Medical Research Council funded study (grant number G0601685). The MSTIC study aims to develop an evidence-based, web-based tool to assist those planning sexual health services for local populations in determining the relative public health impact of different combinations of health services. The web-tool will incorporate the results of a discrete event simulation mathematical model of the key factors influencing the transmission of common STIs so that the effect of different combinations of clinical services on averting transmission can be assessed. The model will use publicly-available data about local populations, for example: local census data and routinely-collected surveillance such as the GUM Clinic Activity Dataset (GUMCAD)[9] and GUM Access Monthly Monitoring (GUMAMM) data.[10] However, while GUMCAD and GUMAMM can provide basic sociodemographic data as well as data on STI testing and positivity for patients attending GUM clinics and increasingly, primary-care based LESSH services, the range of relevant information collected by these surveillance systems is limited.

We are therefore developing a survey tool in the form of a patient questionnaire that can be linked to an extract of patients' clinical records for clinical services to use to provide much more insight into their local patient populations, including questions on their care pathways and transmission-risk behaviours. Data collected by the survey tool can then be used to inform evidence-based decisions about service configuration either via the MSTIC web-tool, or independently, in the context of audits and/or service evaluation, enabling data to be gathered that are comparable over time and between services. This paper now describes the development of the survey tool in GUM clinics and primary-care based services.

### Ethics

The survey tool is being developed in a research context and as such has required Research Ethics approval, which was obtained from the London Research Ethics Committee (reference: 09/H0718/1). However, the tool is intended for routine use in audit and service development, which do not currently require ethical review in the UK[11,12].

## Methods/Design

### Design

We are using a quantitative, cross-sectional survey design to collect data via a pen-and-paper questionnaire from patients attending clinical services offering care for STIs. Data from patients' questionnaires are then linked, with patients' consent, to an extract of their routinely-collected clinical data by means of patients' clinical identifiers.

### Settings

Three sociodemographically- and geographically-contrasting areas in England, broadly representing urban, suburban and rural populations, have been purposively selected to implement the survey tool.

### Study population

The study population is defined as patients attending four principal GUM clinics and, in the rural area, a further four satellite clinics operating from the main GUM clinic on a weekly or fortnightly basis. Patients attending three general practices operating as LESSHs by each holding a weekly sexual health clinic session in the rural area, are also being surveyed.

Acknowledging that young people are a particular 'risk group' for poor sexual health[13,14] we have decided to include teenagers so that we can capture the needs, access patterns and services received by this important minority of service users. However, we are excluding children aged 13 and under as they form a very small minority of sexual health service users, for which ethical issues around informed consent to participate in research are likely to outweigh the advantages gained through including the likely small number of patients of this age.

Study materials will only be available in English as the participating services report a high level of literacy in English in their non-UK born patients, and also due to feasibility constraints.

### Implementation

Reception staff at the services are to offer questionnaires to patients upon arrival at reception and to mark each questionnaire with the date of attendance and the patient's clinical identifier. In the GUM services, questionnaires are for all patients, whereas in the LESSH services run in general practices, questionnaires are administered during sexual health clinic sessions, meaning that sexual health patients can be identified without asking their reason for attendance.

All patients are asked to tick a box on the first page of the questionnaire to indicate consent to our linking their questionnaire data to an extract of their clinical records. Upon the study co-ordinator (CA) receiving completed questionnaires, the services will be provided with a list of the clinical identifiers and dates of attendance of consenting patients. Depending on the service's preference, services will either provide an electronic extract of the relevant data from their clinical database, or will be provided with paper forms or spreadsheets in which to record extracts of the clinical data of consenting patients.

### Development of data collection instruments

The data collected by the questionnaire include basic sociodemographics, reasons for attendance, duration of care-seeking and any experience of other services, recent sexual history (including sex since recognising a need to seek care), and presence and duration of symptoms. The questionnaire is based on that used by a previous study,[15] enhanced to include questions about recent sexual partnerships, adapted from the forthcoming third British National Survey of Sexual Attitudes and Lifestyles (Natsal-3),[16] a national probability survey of sexual behaviour. In particular, we are seeking to distinguish partnerships that have ended from those that are ongoing when the patient attends the service, as this has implications for the likelihood of successful partner notification.[17] Data on STI tests performed, STI diagnoses made, and partner notification outcomes will be obtained from the clinical records of consenting patients. A full list of data items, including the rationale for collecting each item, and its source by service type, is presented in Table 1.

**Table 1:** Data items collected by the survey tool by source and service type and rationale for collection.

Source	Item	Rationale*
Questionnaire *and *clinical data extract	Gender, age (date of birth and date of attendance used to calculate age in years from clinical data extract)	To find out the demographic profile served by the clinic/LESSH practice. Prevalence of STIs varies by age and gender. Collect from both the questionnaire and clinical data extract in order to check the correct match has been made.

Questionnaire	Reason for attendance	Comparing reason for attendance with the patient's care pathway (duration, any other services used) can be informative. For instance, patients attending because of their own symptoms, or because a partner has been diagnosed with an STI, should ideally be accessing services fast. This also informs the proportion of patients attending due to clinic- or patient-led partner notification (i.e. people attending for new episodes of care who report that they were called in by the clinic, or that their partner has been diagnosed with an infection, as reasons for attendance).
	
	Duration of care-seeking	This can help identify groups of patients for whom access is difficult (i.e. lengthy care-seeking). If the patient has an STI, delays in care-seeking (and therefore time spent infectious) can influence the progress of the disease, and the likelihood of transmission (depending on sexual behaviour).
	
	Use of other services (and if relevant, type/name of service, how long ago and what happened)	We can use this information to assess whether there has been duplication of effort in a patient's care pathway, and/or whether their STI could have been detected and treated by the previous service(s) they contacted. We will also be able to see which services refer patients on (and assess whether this is likely to be justifiable). (Proviso: obviously we will not have data from patients who abandoned care-seeking and so were never surveyed).
	
	Number of male/female sexual partners in the last year; Number of these who were *new *partners; Number of partners in the last 3 months;	Number and gender of recent partners influences STI risk. It is also informative to find out whether people with many partners, and men who have sex with men, are more likely to attend particular services.
	
	Regarding each partner in the last 3 months (up to a max. of 3): - how long ago first had sex, - how long ago most recently had sex, - condom use, - expectation of having sex again with this person;	How long ago each sexual partnership began and ended informs measurement of concurrency (partnership overlap), which influences transmission risk. Expectation of having sex with a person again informs measurement of concurrency (above) and is an indicator of the likelihood of successful partner notification. Condom use informs the extent to which the partnership was 'protected' and (if low) suggests in which groups within the service's users further health promotion on condom use might be needed
	
	Whether the respondent had sex since recognising a need to seek care (for the reason attending the service), and if so: - number of partners, - number of new partners, - number of occasions of sex, - condom use	Together with STI diagnosis this informs the likelihood of transmission since the patient recognised the need to seek care. This information can be compared with patients' reason(s) for attendance, presence of symptoms, and STIs diagnosed. It may indicate a need for health promotion messages about abstaining from sex once the need to seek testing/care is recognised.
	
	Whether the respondent has ever been diagnosed with an STI	Past STI diagnosis may affect future care-seeking.
	
	Whether the respondent has ever had a *Chlamydia *test, and if so in what setting	A measure of past contact with *Chlamydia *screening services. The care-seeking and demographics of patients who have tested for *Chlamydia *before, and specifically at a sexual health clinic, can be compared to those who have not.
	
	Whether the respondent has symptoms now, and if so, duration of symptoms	Although many infections are asymptomatic, it is useful to know how patients with symptoms differ in their care-seeking to those without.
	
	Whether the respondent is registered with a GP	Patients registered with a GP may have more opportunity to seek sexual health care from primary care (including LESSH) than those not registered.
	*LESSH only:* - whether the patient attended for a booked appointment or as a 'walk-in' patient - whether the LESSH GP is the patient's own GP - and if not, the name of the patient's GP surgery	The type of appointment can influence the length of time a patient waits (in contrast we know that once they contact a clinic, most GUM patients are seen within 48 hours) Patients registered with a practice providing a LESSH may be more likely to attend the LESSH. It is also important to know whether patients not registered at a LESSH practice benefit from the service, and if so how many.

Clinical data extract	STIs tested for; STIs diagnosed;	Knowing which STIs were tested for informs which STIs *could have been *diagnosed. Patients with STIs can be compared to patients without STIs, by demographics, reason(s) for attendance, care pathway duration, etc.
	
	Whether patient is already known to be HIV positive	As HIV testing is not relevant for patients known to be HIV positive
	
	Partner notification outcomes for patients diagnosed with *Chlamydia *or gonorrhoea (at least one partner tested; at least one partner treated)	These indicators of partner notification assess the extent to which the service is managing to notify the partners of patients with common STIs.
	
	*LESSH only:* - whether microscopy was performed; - treatment received - referral to other services	We know that GUM services have facilities for microscopy but some LESSH may not have this facility. Similarly, we wished to measure the appropriateness of treatment received by doctors whose main work is *not *sexual health. We also wanted to measure how commonly patients were referred on for problems that *could *have been dealt with by the LESSH (for instance if practices or contracts changed), particularly referral to GUM.
	
	*GUM only:* - PCT of residence	As LESSH are advertised within a PCT, we assumed the majority of patients lived in the same PCT. GUM clinic users may travel from further afield; data on PCT of residence is routinely collected in GUM.

Source differed by setting	Collected in clinical data extract in GUM but questionnaire in LESSH: - ethnicity; - new/follow-up attendance status; - past attendance at the service *(in LESSH services patients were asked to state whether this was for a sexual health or other primary care reason, or both)*; - name of GP surgery	Ethnicity is routinely by GUM clinics, but not necessarily by LESSH services. STI prevalence varies by ethnicity, and ethnicity can be used to assess whether any groups are underserved, attend for different reasons, or have longer care pathways. GUM clinics routinely code whether patients are attending as new patients, rebook (i.e. patient attending for a new episode of care, but who has attended before), and follow-up (subsequent visits in the same episode of care). We gained this information from the questionnaire for LESSH patients, and additionally asked whether the patient had attended the practice for a reason not related to sexual health. GUM clinics routinely ask patients their GP (though patients are not required to provide it), whereas this data may not be routinely collected by LESSH services. We could assess from this what PCT the patient's GP was in (which sometimes differed from PCT of residence), and whether the practice provided a LESSH. It is interesting to know whether patients whose own GP surgery provides a LESSH chose to attend this service or GUM.

*For our study, the information on patients' demographics, sexual behaviour, and STI positivity also informs a mathematical model of STI transmission. Here we concentrate on the rationale for the collection of audit data for local use.

Though collecting comparable data, the questionnaire and clinical data extract differ slightly between GUM and LESSH services, reflecting differences in the nature of the services and in the patient data routinely collected for surveillance purposes. As such, LESSH patients will be asked a different question about their past use of the service to distinguish between attending the practice for sexual health reasons versus other reasons. If registered with a GP, these patients will be asked which practice, for the purpose of determining whether they are registered with the LESSH practice they are attending, another LESSH service, or a practice which does not provide this service. Recognising that staff in general practice are not primarily sexual health specialists, the LESSH data extract includes treatment administered, while we assume that clinical guidelines (e.g. [18,19]) are followed in GUM clinics. GUM clinical data extracts include patients' PCT of residence as people often attend clinics outside their PCT of residence, especially within London[20]. There are some further minor differences in questionnaire and clinical data extract wording between versions - see Additional files 1, 2, 3, 4, 5 and 6 for the questionnaires and the paper proforma for clinical data extracts for use by each service type.

We carried out a two-stage pilot to test the questionnaire. Thirteen cognitive interviews with patients at one of the participating GUM clinics were undertaken to assess whether the survey tool was acceptable to patients, whether questions were understood and answered as the research team intended, and whether response options were adequate. Data on the sociodemographic characteristics of these patients are provided in Table 2 and were considered by clinic staff to be broadly representative of their clinic population. The second stage involved piloting the questionnaire over a two-day period at the same clinic, which resulted in 56 out of 81 patients completing the questionnaire, i.e. a 67% response rate, of whom 84% consented to linkage. Questionnaires in this pilot were completed with low item non-response, very few inconsistent answers, and instances where the respondent had not followed the routing were minimal.

**Table 2:** Sociodemographic characteristics of patients participating in cognitive interviews

Characteristic ^1^		Number of patients	Source of data
Gender	Female	7	Self-reported in questionnaire
		
	Male	6	
	
Age	15-19	1	
		
	20-24	1	
		
	25-29	4	
		
	30-34	4	
		
	35-39	1	
		
	40-45	2	
	
Sexuality^2^	Heterosexual	9	
		
	Homosexual	1	
		
	Bisexual	2	
		
	Not asked	1	

Ethnicity	White	9	Assessed by interviewer^3^
		
	Black	2	
		
	Asian	2	

Other information	English not main language	2	Spontaneous, self-reported
		
	'My reading's rubbish' (native-speaker)	1	

The demographic breakdown was considered by clinic staff to be broadly representative of their clinic population. However, it is worth noting that patients at this clinic tended to be slightly older and more likely to be from professional backgrounds than patients attending the study's other GUM clinics but for logistical reasons it was only feasible to undertake the cognitive interviews in one GUM clinic.

Based on reported gender of sexual partner(s) in the last year.

Assessed by the interviewer (CA) for the cognitive interview data only. During survey implementation, LESSH patients self-reported their ethnicity, and GUM patients' ethnicity was obtained later from the clinical data extract.

### Patient confidentiality

Precautions taken to protect patient confidentiality include: not asking for patients' names, addresses or signature at any point - instead asking patients to tick a box to indicate consent; providing locked boxes in reception areas so that other patients do not have access to completed questionnaires; and providing envelopes in which patients seal their questionnaires, so that patients can conceal their questionnaire if they move around the clinic and so that clinic staff outside of the research team do not see completed questionnaires. The questionnaire data and clinical data extracts will be pseudonymous as both datasets contained patient clinical identifiers, necessary for linkage. Nonetheless the databases will be securely stored in password-protected files and after linking the datasets, the clinical identifiers will be deleted, rendering the data anonymous.

### Data collection periods

Data collection is likely to take from three to ten weeks per service, according to the likely numbers of patients attending the service and anticipated response rates, based on experience from an earlier study.[15] Sample size calculations are not considered necessary, reflecting the aim of gathering data *rapidly *to inform service planning, and that use of the survey tool should, as an audit of the patient populations attending services, provide *indicative *data, such that the attainment of large numbers will not be necessary.

### Data entry and analysis

Questionnaire data and clinical data recorded on paper forms will be double-entered into a Microsoft Access database by study administrators (estimated to take approximately five minutes per questionnaire, two minutes per data extract). These data will then be transferred into the statistical package STATA version 10[21] for statistical analysis. However, users of the survey tool without access to a specialist statistical package will be able to use the query functions in Microsoft Access to generate descriptive statistics. Any sub-group analyses with denominators of less than 25 will need to be treated with caution and 95% confidence intervals will be reported with all estimates to reflect the effect of the size of the achieved sample.

## Discussion

To our knowledge, this will be the first study to collect comparable, detailed data from specialist GUM clinics *and *primary-care based LESSH services. To date, little has been known about the characteristics and behaviours of patients attending new models of sexual health service provision, such as LESSH. Our survey tool will provide a feasible and rapid means of collecting data from diverse clinical services, including these highly variable[4,5] models of sexual health service provision.

We anticipate satisfactory response rates under the conditions in which clinical services are likely to use the survey tool for planning purposes: researchers will not be present to administer the questionnaire, there will be no incentives or tokens of appreciation for patients or reception staff, and the questionnaires cover some highly-sensitive topics. The response rate for the pilot study was similar to the 65.4% response rate achieved by the last British probability survey of sexual behaviour (the second National Survey of Sexual Attitudes and Lifestyles),[22,23] a researcher-intensive study. However, there may be considerable variation in response rates between services, which was observed in the earlier study and attributed to enthusiasm for the research varying between reception staff teams, in some services reflecting short-staffing.[15]

We will only be able to administer the LESSH survey tool in one of the three study areas for reasons of feasibility and appropriateness: one area currently does not have a LESSH and in the other, patients seeking care for suspected STI are currently seen within the general surgery lists and we feel that it will be inappropriate for receptionists to identify these patients by asking them their reasons for attendance. However, should such services wish to implement the survey tool then the questionnaires could be distributed during patients' consultations, requiring reliance upon the co-operation and memory of GPs and practice nurses instead of reception staff.

Ensuring enthusiasm from those staff charged with distributing the questionnaire is expected to be important in terms of achieving both a high response rate from patients and a high rate of accurate completion of patients' clinical identifiers and dates of attendance on the front of the questionnaires. While we anticipate a high proportion of respondents will consent to our linking their questionnaire data to their clinical data, in a previous study[15] this was not possible for a handful of cases as clinical identifiers and/or dates were illegible, missing, or the clinical identifier did not correspond to an attendance on the date given. Aside from time, the enthusiasm and organisational skills of key individuals, such as reception supervisors, is likely to be vital to the successful administration of the survey tool.

Clinical services that have used electronic patient records for some time should be able to provide the research team with their clinical data extracts quickly. However, for services that still rely on paper notes this is likely to be a time-intensive process. Obtaining the clinical data extract will become quicker and easier as all clinical services move towards electronic patient records systems, and this will further facilitate the use of the survey tool.

In conclusion, development work for our survey tool suggests that it will be a rapid and easy method for sexual health services to collect detailed, individual-level data on their patients, supplementing existing surveillance data to better understand their patient populations and their care pathways so that services can be made more clinically- and cost-effective. Our paper's additional files will allow service providers and planners to implement the survey tool themselves, which will be feasible with enthusiastic staff, without the need for researchers to be present or additional resources.

## List of abbreviations used

GUM - genitourinary medicine, HIV - human immunodeficiency virus, LESSH - Local Enhanced Service (sometimes known as 'LES') for sexual health, MSTIC - Maximising Sexually Transmitted Infection Control (the study's short title), STI - sexually transmitted infection(s)

## Competing interests

The authors declare that they have no competing interests.

## Authors' contributions

JC, FK and CM conceived the study; all authors are participating in its design; CM is responsible for the overall study; CA is responsible for securing the necessary permissions and will be managing the data collection from all services; CE, GB and FK will be managing data collection at their GUM clinics; CA and CM drafted the paper with input from all authors. All authors read and approved the final manuscript.

## Pre-publication history

The pre-publication history for this paper can be accessed here:

http://www.biomedcentral.com/1472-6963/11/30/prepub

## Supplementary Material

Additional file 1 **The patient questionnaire administered in GUM clinics**. Click here for file

Additional file 2 **The patient questionnaire administered in GUM clinics**. Click here for file

Additional file 3 **The patient questionnaire administered in LESSH practices**. Click here for file

Additional file 4 **The patient questionnaire administered in LESSH practices**. Click here for file

Additional file 5 **Clinical data extract form used for GUM clinic patients**. Click here for file

Additional file 6 **Clinical data extract form used for LESSH patients**. Click here for file
