# Supplementary material for: Rationale and development of a survey tool for describing and auditing the composition of, and flows between, specialist and community clinical services for sexually transmitted infections
Source: BMC Health Serv Res. 2011 Feb 9;11:30. doi: 10.1186/1472-6963-11-30 (PMC3045289; doi:10.1186/1472-6963-11-30)
Supplement: Additional file 1 — The patient questionnaire administered in GUM clinics. [file 1472-6963-11-30-S1.PDF]

Patient Survey

Date: | | | | | | No. |

If you fill in this questionnaire, you are agreeing that the researchers can ask the nurse or doctor you are seeing today for some information about the reason you are attending. The researchers will not know who you are, but will get information about what tests you had, your diagnosis (e.g. 'warts' or 'Chlamydia'), and treatment and action taken. They will tell us if you are a new patient, the name of your GP surgery, your ethnic group and also your date of birth and gender. The clinic will **not** give us your name or address, we will match the information using a number instead. The clinic staff will **not** see what you write on this questionnaire.

We are not asking you to sign your name to show you agree, because we don't need to know what your name is. But we do want to be sure that you understand that we will ask the clinic for this information.

1. Please tick this box if you agree to us getting this information ☐

2. Are you:

female ☐
male ☐

3. How old are you? years

4. Have you been to <u>this clinic</u> in the past?

Yes, a while ago ☐
Yes, and I'm here for a follow-up, today ☐
No, I have never been here before ☐

Thank you very much for taking the time to complete this questionnaire

If there is anything you want to ask about this study, please contact:

This is the last page of the questionnaire

17. Have you ever, in the past, been diagnosed with a sexually transmitted infection (STI)?

Yes
I'm not sure
No

18. Have you ever had a Chlamydia test?

No
I'm not sure
Yes

If yes, where?

At a pharmacy / chemists
At a GP surgery
At a "GUM clinic" (a clinic like this one)
At a Family Planning Clinic
At a youth group or sports club
At a different place *(please tell us where)*

19. Do you have symptoms now? (e.g. pain or discharge)

No
I'm not sure
Yes

If yes, how long ago did these symptoms start?

My symptoms started today

or My symptoms started days ago
or weeks ago

20. Are you registered with a GP?

No
I'm not sure
Yes

Thinking about the reason why you came here today:

5. Why did you come to this clinic? *(You can tick more than one)*

I have (or had) symptoms (e.g. itching, discharge)
My partner has (or had) symptoms
I did not have symptoms but wanted a check-up
My partner has been diagnosed with an infection and I needed to come to the clinic
Someone from the clinic called me in
I wanted to have an HIV test
My GP or practice nurse told me to come here
My symptoms have not gone away since I last came here for treatment
Last time I came here someone asked me to come back for more treatment, or for another check-up / test
For a different reason *please tell us about this:*

6. When did you first try to contact any clinic, GP or other health professional, about the reason you came here today?

Today
Before today *please say how many days ago:*

days ago

I just walked in today

7. Before coming here today, did you use or try to use any other healthcare services for treatment or advice, for the problem you have today? *(This could be in person, by phone or on the internet)*

No *please go to question 9*
Yes *please continue to question 8*

If you change your mind about the survey, please cross out or tear the first page, put the survey in the envelope, and put it in the box. The researchers will destroy it confidentially, and the clinic will not give them any information about you.

8. Which other services did you use or try to use **for the problem you have today?**

8a. A GP surgery

No, I did not use or try to use a GP surgery ☐ *please go to question **8b***

Yes, I used (or tried to use) a GP surgery ☐ *please continue*

How long ago?

_______ days ago **or** _______ weeks ago

Please tell us which GP surgery you used (or tried to use):

____

What happened at that GP surgery? *(you can tick more than one box)*

- I tried to contact the surgery, but didn't manage to get through ☐
- I went there in person ☐
- I couldn't get an appointment soon enough ☐
- I couldn't get an appointment at a convenient time ☐
- I saw a GP or nurse ☐
- I was given treatment ☐
- I was given a prescription to collect treatment from a pharmacy ☐
- I took the treatment I was given or prescribed ☐
- I was advised to attend a GUM clinic *(this clinic or a clinic like this one)* ☐
- I was advised to go somewhere else ☐ *please tell us where:*

____

Other ☐ *please tell us what happened*

____

If you change your mind about the survey, please cross out or tear the first page, put the survey in the envelope, and put it in the box. The researchers will destroy it confidentially, and the clinic will not give them any information about you.

16a. How many people have you had sex with since you thought you might need to go to a clinic or GP surgery for the reason you came here today?

____

16b. How many of these people were new partners? *(people you first had sex with since you thought you might need to go to a clinic or GP surgery)*

____

16c. Since you thought you might need to go to a clinic or GP surgery, approximately how many times have you had sex? *(with all partners)*

__________ *times*

16d. Since you thought you might need to go to a clinic or GP surgery, how often have you used condoms when you had sex? *(with all partners)*

*please tick **one** box:*

- Not at all ☐
- Sometimes (about ¼ of the time) ☐
- Half of the time ☐
- Most times (about ¾ of the time) ☐
- Every time ☐

These questions are about sex since you thought you might need to go to a clinic or GP surgery, for the reason you came here today.

15. Since you thought you might need to go to a clinic or GP surgery, have you had sex?

No ☐ *please go to question **17***
Yes ■ *please continue to question **16***

If you change your mind about the survey, please cross out or tear the first page, put the survey in the envelope, and put it in the box. The researchers will destroy it confidentially, and the clinic will not give them any information about you.

8b. Another service

No, I did not use or try to use any other service ■ *please go to question **9***

Yes, I used (or tried to use) another service ☐ *please continue*

How long ago?

_______ days ago **or** _______ weeks ago

What was that service called?

____

What happened? *(you can tick more than one box)*

I tried to contact them, but didn't manage to get through ☐
I went there in person ☐
I couldn't get an appointment soon enough ☐
I couldn't get an appointment at a convenient time ☐
I saw a doctor or nurse ☐
I was given treatment ☐
I was given a prescription to collect treatment from a pharmacy ☐
I took the treatment I was given or prescribed ☐
I was advised to attend a GUM clinic *(this clinic or a clinic like this one)* ☐
I was advised to go to my own GP ☐
I was advised to go somewhere else ☐ *please tell us where:*

Other ☐ *please tell us what happened*

These questions are about you and your partner (or partners):

9. In the last 12 months (since this time last year), how many people have you had sex with?

Number of women: ______ Number of men: ______

10. How many of these people were new partners who you had sex with for the first time during the last 12 months?

Number of women: ______ Number of men: ______

11. In the last 3 months, how many people have you had sex with?

____

The next questions are about your most recent sexual partner or partners, in the last 3 months. If you have not had sex in the last 3 months, please go to question 15.

Thinking about the person you most recently had sex with:

12a. When did you first have sex with this person?

please tell us how long ago __________ days ago
or __________ weeks ago
or __________ months ago
or __________ years ago

12b. When did you most recently have sex with this person?

please tell us how long ago __________ days ago
or __________ weeks ago
or __________ months ago

12c.Many people find it difficult to use condoms consistently. How often did you use condoms when you had sex with this person?

Not at all ☐ The first few times ☐ Almost every time ☐ Every time ☐

12d. Do you expect to have sex with this person again?

No ☐ Probably not ☐ I don't know ☐ Probably ☐ Yes ☐

If you have had sex with just this one person in the last 3 months, please go to question 15. If you have had sex with more than one person in the last 3 months, please continue.

If you change your mind about the survey, please cross out or tear the first page, put the survey in the envelope, and put it in the box. The researchers will destroy it confidentially, and the clinic will not give them any information about you.

Thinking about your second most recent sexual partner:

13a. When did you first have sex with this person?

please tell us how long ago: __________ days ago
or __________ weeks ago
or __________ months ago
or __________ years ago

13b. When did you most recently have sex with this person?

please tell us how long ago: __________ days ago
or __________ weeks ago
or __________ months ago

13c.Many people find it difficult to use condoms consistently. How often did you use condoms when you had sex with this person?

Not at all ☐ The first few times ☐ Almost every time ☐ Every time ☐

13d. Do you expect to have sex with this person again?

No ☐ Probably not ☐ I don't know ☐ Probably ☐ Yes ☐

If you have had sex with just these 2 people in the last 3 months, please go to question 15. If you have had sex with more than 2 people in the last 3 months, please continue.

Thinking about your third most recent sexual partner:

14a. When did you first have sex with this person?

please tell us how long ago __________ days ago
or __________ weeks ago
or __________ months ago
or __________ years ago

14b. When did you most recently have sex with this person?

please tell us how long ago __________ days ago
or __________ weeks ago
or __________ months ago

14c.Many people find it difficult to use condoms consistently. How often did you use condoms when you had sex with this person?

Not at all ☐ The first few times ☐ Almost every time ☐ Every time ☐

14d. Do you expect to have sex with this person again?

No ☐ Probably not ☐ I don't know ☐ Probably ☐ Yes ☐
