# Supplementary material for: Rationale and development of a survey tool for describing and auditing the composition of, and flows between, specialist and community clinical services for sexually transmitted infections
Source: BMC Health Serv Res. 2011 Feb 9;11:30. doi: 10.1186/1472-6963-11-30 (PMC3045289; doi:10.1186/1472-6963-11-30)
Supplement: Additional file 3 — The patient questionnaire administered in LESSH practices. [file 1472-6963-11-30-S3.PDF]

Patient Survey

Date: | | | | | | No. |

If you fill in this questionnaire, you are agreeing that the researchers can ask the nurse or doctor you are seeing today for some information about the reason you are attending. The researchers will not know who you are, but will get information about what tests you had, your diagnosis (e.g. 'warts' or 'Chlamydia'), and treatment and action taken. They will also tell us your date of birth and gender. The clinic will **not** give us your name or address, we will match the information using a number instead. The GP surgery staff will **not** see what you write on this questionnaire.

We are not asking you to sign your name to show you agree, because we don't need to know what your name is. But we do want to be sure that you understand that we will ask the GP surgery for this information.

1. Please tick this box if you agree to us getting this information ☐

2. Are you:

female ■

male ☐

3. How old are you? years

4. Please tell us which ethnic group you belong to:

Asian or Asian British	☐	White or White British	☐
Black or Black British	■	Other ethnic group	☐
Mixed ethnicity	☐	Prefer not to say	☐

Thank you very much for taking the time to complete this questionnaire

If there is anything you want to ask about this study, please contact:

This is the last page of the questionnaire

23. Have you ever, in the past, been diagnosed with a sexually transmitted infection (STI)?

- Yes
- I'm not sure
- No

24. Have you ever had a Chlamydia test?

- No
- I'm not sure
- Yes

If yes, where?

- At a pharmacy / chemists
- At a GP surgery
- At a "GUM clinic" (a specialist sexual health clinic)
- At a Family Planning Clinic
- At a youth group or sports club
- At a different place *(please tell us where)*

25. Do you have symptoms now? (e.g. pain or discharge)

- No
- I'm not sure
- Yes

If yes, how long ago did these symptoms start?

My symptoms started today

or My symptoms started days ago

or weeks ago

5. Have you been to <u>this GP surgery</u> in the past?

- No, I have never been here before *please continue to question **6***
- Yes

Was it for a reason to do with sexual health?

- Yes, and it was a while ago
- Yes, and I'm here for a follow-up, today
- No, it was for a different reason
- I have been here for sexual health and other reasons

6. Are you registered with a GP?

- No *please go to question **8** on the next page*
- I'm not sure *please continue to question **7***
- Yes *please continue to question **7***

7. Is this your GP surgery?

- Yes *please continue to the next page*
- I'm not sure *please continue to the next page*
- No

Please tell us the name of your GP surgery, so that we can find out whether it provides a special sexual health service

Name of GP surgery:

If you change your mind about the survey, please cross out or tear the first page, put the survey in the envelope, and put it in the box. The researchers will destroy it confidentially, and the GP surgery will not give them any information about you.

Thinking about the reason why you came here today:

8. Why did you come to the GP surgery? *(You can tick more than one)*

- I have (or had) symptoms (e.g. itching, discharge) ☐
- My partner has (or had) symptoms ☐
- I did not have symptoms but wanted a check-up ☐
- My partner has been diagnosed with an infection and I needed to come here ☐
- Someone from the surgery called me in ☐
- I wanted to have an HIV test ☐
- My GP or practice nurse told me to come here ■
- My symptoms have not gone away since I last came here for treatment ☐
- Last time I came here someone asked me to come back for more treatment, or for another check-up / test ☐
- For a different reason ☐ *please tell us about this:*

____

If you change your mind about the survey, please cross out or tear the first page, put the survey in the envelope, and put it in the box. The researchers will destroy it confidentially, and the GP surgery will not give them any information about you.

22a. How many people have you had sex with since you thought you might need to go to a clinic or GP surgery for the reason you came here today?

____

22b. How many of these people were new partners? *(people you first had sex with since you thought you might need to go to a clinic or GP surgery)*

____

22c. Since you thought you might need to go to a clinic or GP surgery, approximately how many times have you had sex? *(with all partners)*

__________ *times*

22d. Since you thought you might need to go to a clinic or GP surgery, how often have you used condoms when you had sex? *(with all partners)*

*please tick **one** box:*

- Not at all ☐
- Sometimes (about ¼ of the time) ☐
- Half of the time ☐
- Most times (about ¾ of the time) ☐
- Every time ■

These questions are about sex <u>since</u> you thought you might need to go to a clinic or GP surgery, <u>for the reason you came here today.</u>

21. Since you thought you might need to go to a clinic or GP surgery, have you had sex?

No ☐ *please go to question **23***
Yes ☐ *please continue to question **22***

9. When did you <u>first</u> try to contact <u>any</u> clinic, GP or other health professional, about the reason you came here today?

Today ☐
Before today ☐ *please say how many days ago:*

days ago

I just walked in today ☐

10. Before coming here today, did you use or try to use <u>any other healthcare services</u>, for treatment or advice for the problem you have today? *(This could be in person, by phone or on the internet)*

No ☐ *please go to question **12***
Yes ☐ *please continue to question **11***

If you change your mind about the survey, please cross out or tear the first page, put the survey in the envelope, and put it in the box. The researchers will destroy it confidentially, and the GP surgery will not give them any information about you.

11. Which other services did you use or try to use <u>for the problem you have today?</u>

11a. Another GP surgery (not this one)

No, I did not use or try to use any other GP surgery ☐ *please go to question **11b***

Yes, I used (or tried to use) another GP surgery ☐ *please continue*

How long ago?

_______ days ago **or** _______ weeks ago

Please tell us which GP surgery you used (or tried to use):

____

What happened at that GP surgery? *(you can tick <u>more than one</u> box)*

I tried to contact the surgery, but didn't manage to get through ☐

I went there in person ☐

I couldn't get an appointment soon enough ☐

I couldn't get an appointment at a convenient time ☐

I saw a GP or nurse ☐

I was given treatment ☐

I was given a prescription to collect treatment from a pharmacy ☐

I took the treatment I was given or prescribed ☐

I was advised to attend a 'GUM' clinic *(a specialist sexual health clinic)* ☐

I was advised to go to this GP surgery ■

I was advised to go somewhere else ☐ *please tell us where:* ▼

____

Other ■ *please tell us what happened* ☐

____

If you change your mind about the survey, please cross out or tear the first page, put the survey in the envelope, and put it in the box. The researchers will destroy it confidentially, and the GP surgery will not give them any information about you.

Thinking about your <u>second most recent</u> sexual partner:

19a. When did you first have sex with this person?

please tell us how long ago: __________ days ago
or __________ weeks ago
or __________ months ago
or __________ years ago

19b. When did you most recently have sex with this person?

please tell us how long ago: __________ days ago
or __________ weeks ago
or __________ months ago

19c.Many people find it difficult to use condoms consistently. How often did you use condoms when you had sex <u>with this person</u>?

Not at all ☐ The first few times ☐ Almost every time ☐ Every time ☐

19d. Do you expect to have sex with this person again?

No ☐ Probably not ☐ I don't know ☐ Probably ☐ Yes ☐

If you have had sex with just these 2 people <u>in the last 3 months</u>, please go to question <u>21</u>. If you have had sex with more than 2 people in the last 3 months, please continue.

Thinking about your <u>third most recent</u> sexual partner:

20a. When did you first have sex with this person?

please tell us how long ago __________ days ago
or __________ weeks ago
or __________ months ago
or __________ years ago

20b. When did you most recently have sex with this person?

please tell us how long ago __________ days ago
or __________ weeks ago
or __________ months ago

20c.Many people find it difficult to use condoms consistently. How often did you use condoms when you had sex <u>with this person</u>?

Not at all ☐ The first few times ☐ Almost every time ☐ Every time ☐

20d. Do you expect to have sex with this person again?

No ☐ Probably not ☐ I don't know ☐ Probably ☐ Yes ☐

These questions are about you and your partner (or partners):

15. In the last 12 months (since this time last year), how many people have you had sex with?

Number of women: ______ Number of men: ______

16. How many of these people were new partners who you had sex with for the first time during the last 12 months?

Number of women: ______ Number of men: ______

17. In <u>the last 3 months</u>, how many people have you had sex with?

____

The next questions are about your most recent sexual partner or partners, <u>in the last 3 months</u>. If you have not had sex in the <u>last 3 months, please go to question 21.</u>

Thinking about the person you <u>most recently</u> had sex with:

18a. When did you first have sex with this person?

please tell us how long ago __________ days ago
or __________ weeks ago
or __________ months ago
or __________ years ago

18b. When did you most recently have sex with this person?

please tell us how long ago __________ days ago
or __________ weeks ago
or __________ months ago

18c.Many people find it difficult to use condoms consistently. How often did you use condoms when you had sex <u>with this person</u>?

Not at all ☐ The first few times ☐ Almost every time ☐ Every time ☐

18d. Do you expect to have sex with this person again?

No ☐ Probably not ☐ I don't know ☐ Probably ☐ Yes ☐

If you have had sex with just this one person in the last 3 months, please go to question <u>21</u>. If you have had sex with more than one person in the last 3 months, please continue.

If you change your mind about the survey, please cross out or tear the first page, put the survey in the envelope, and put it in the box. The researchers will destroy it confidentially, and the GP surgery will not give them any information about you.

11b. Another service

No, I did not use or try to use any other service ☐ *please go to question **12***

Yes, I used (or tried to use) another service ☐ *please continue*

How long ago?

days ago **or** weeks ago

What was that service called?

____

What happened? *(you can tick <u>more than one</u> box)*

I tried to contact them, but didn't manage to get through ☐
I went there in person ☐
I couldn't get an appointment soon enough ☐
I couldn't get an appointment at a convenient time ☐
I saw a doctor or nurse ☐
I was given treatment ☐
I was given a prescription to collect treatment from a pharmacy ☐
I took the treatment I was given or prescribed ☐
I was advised to attend a 'GUM' clinic *(a specialist sexual health clinic)* ☐
I was advised to go to this GP surgery ☐
I was advised to go to my own GP ☐
I was advised to go somewhere else ☐ *please tell us where:*

Other ☐ *please tell us what happened*

We would like to know how easy it was for you to make an appointment here

12. Did you walk in today or have a booked appointment?

I booked an appointment before coming here today

*please go to question **13***

I walked in today without an appointment

*please go to question **14***

13. When did you first contact this GP surgery to make your appointment for today?

I contacted the GP surgery today to make this appointment

I contacted the GP surgery [ ] days ago to make this appointment

I made this appointment last time I came

*please go to question **15** on the next page*

14. Did you contact this GP surgery before walking in today?

No

Yes, today

Yes, before today **How long ago?** [ ] days ago

*please go to question **15** on the next page*
