# Supplementary material for: Rationale and development of a survey tool for describing and auditing the composition of, and flows between, specialist and community clinical services for sexually transmitted infections
Source: BMC Health Serv Res. 2011 Feb 9;11:30. doi: 10.1186/1472-6963-11-30 (PMC3045289; doi:10.1186/1472-6963-11-30)
Supplement: Additional file 5 — Clinical data extract form used for GUM clinic patients. [file 1472-6963-11-30-S5.DOCX]

**MSTIC Study clinic data extract form: GUM**

| Our ref: |  | | Patient’s clinic number |  | | |
| --- | --- | --- | --- | --- | --- | --- |
| Date of questionnaire/clinic attendance | | | | **/ /** | | |
| Was this the patient’s first attendance ever? | | | | Y / N | | |
| Was it a ‘new’ or follow-up attendance? | | | | *New / Follow-up* | | |
| Gender *(please circle)* | | | | M / F | | |
| Date of birth (dd/mm/yy) | | | | / / | | |
| PCT of residence  *(please look up using* ***patient’s postcode****)* | | | | PCT(s) | | no postcode  given |
| Patient’s GP surgery, address, postcode (or GP’s name)  *(Please check patient’s file, and top sheet. Please copy even if patient has not given to consent to contact their GP. We will not be contacting individual patients’ GPs).* | | | |  | | |
| Ethnic Origin  *(see top sheet or registration form)* | | | |  | | |
| **Related to the patient’s attendance *on the date in the shaded box*, above:** | | | | | | |
| What tests were done for the patient *on this date?* | | | | | Genital examination **Y / N** | |
|  |  |  |  |  | Chlamydia test **Y / N** | |
|  |  |  |  |  | Gonorrhoea test **Y / N** | |
|  |  |  |  |  | Blood for syphilis **Y / N** | |
|  |  |  |  |  | Blood for HIV **Y / N** | |
|  |  |  |  |  | Other **Y / N** | |
| What diagnoses were made during *this episode of care*?  *(please tick all that apply)* | | **B1/B2/B5** gonorrhoea (complicated or uncomplicated) **Y / N** | | | | |
|  |  | **C4A/C4B/C4C** chlamydial infection (complicated/uncomplicated) **Y / N** | | | | |
|  |  | **C4H**  non-GC/NSU or treatment of mucopurulent cervicitis in females **Y / N** | | | | |
|  |  | **C6A** trichomoniasis **Y / N** | | | | |
|  |  | **C10A** anogenital herpes simplex: first attack **Y / N** | | | | |
|  |  | **C10B** anogenital herpes simplex: recurrence **Y / N** | | | | |
|  |  | **C11A** anogenital warts: first attack **Y / N** | | | | |
|  |  | **C11B** anogenital warts: recurrence **Y / N** | | | | |
|  |  | **A1-A6** syphilis requiring treatment **Y / N** | | | | |
|  |  | **Complicated STI:** *Was patient diagnosed with:* Epididymitis **Y / N** Pelvic inflammatory disease (PID) **Y / N** | | | | |
|  |  | **Newly diagnosed HIV: E1A** New HIV diagnosis: asymptomatic **Y / N**  **E3A1**AIDS: first presentation: new HIV diagnosis **Y / N** | | | | |
|  | | **E1B** Subsequent HIV presentation **Y / N** | | | | |
| Treatment as a contact *on this date*: | | **B4** epidemiological treatment of suspected gonorrhoea **Y / N** | | | | |
|  |  | **C4E** epidemiological treatment of suspected Chlamydia **Y / N** | | | | |
|  |  | **C4I**  epidemiological treatment of suspected NSGI **Y / N** | | | | |
|  |  | **C7B**  epidemiological treatment of trichomoniasis **Y / N** | | | | |
|  |  | **A9** epidemiological treatment of suspected syphilis **Y / N** | | | | |
| **If diagnosed with Gonorrhoea / Chlamydia** (leave blank for patients without GC/CT) | | | | | | |
| At least one partner reported **tested Y / N** | | | | | | |
| At least one partner reported **treated Y / N** | | | | | | |
