# Supplementary material for: Rationale and development of a survey tool for describing and auditing the composition of, and flows between, specialist and community clinical services for sexually transmitted infections
Source: BMC Health Serv Res. 2011 Feb 9;11:30. doi: 10.1186/1472-6963-11-30 (PMC3045289; doi:10.1186/1472-6963-11-30)
Supplement: Additional file 6 — Clinical data extract form used for LESSH patients. [file 1472-6963-11-30-S6.DOCX]

**MSTIC Study clinic data extract form: LES**

| Our ref: |  | | Patient’s ID number |  | |
| --- | --- | --- | --- | --- | --- |
| Date of questionnaire/clinic attendance | | | | / / | |
| Gender *(please circle)* | | | | M / F | |
| Date of birth (dd/mm/yy) | | | | / / | |
| **Related to the patient’s attendance *on the date in the shaded box*, above:** | | | | | |
| What tests were done for the patient *on this date?* | | | | | Genital examination **Y / N** |
|  |  |  |  |  | Chlamydia test **Y / N** |
|  |  |  |  |  | Gonorrhoea test **Y / N** |
|  |  |  |  |  | Blood for syphilis **Y / N** |
|  |  |  |  |  | Blood for HIV **Y / N** |
|  |  |  |  |  | Microscopy (Gram stain) **Y / N** |
|  |  |  |  |  | Other **Y / N** |
| What diagnoses were made during *this episode of care*?  *(please tick all that apply)* | | **B1/B2/B5** gonorrhoea (complicated or uncomplicated) **Y / N** | | | |
|  |  | **C4A/C4B/C4C** chlamydial infection (complicated/uncomplicated) **Y / N** | | | |
|  |  | **C4H**  non-GC/NSU or treatment of mucopurulent cervicitis in females **Y / N** | | | |
|  |  | **C6A** trichomoniasis **Y / N** | | | |
|  |  | **C10A** anogenital herpes simplex: first attack **Y / N** | | | |
|  |  | **C10B** anogenital herpes simplex: recurrence **Y / N** | | | |
|  |  | **C11A** anogenital warts: first attack **Y / N** | | | |
|  |  | **C11B** anogenital warts: recurrence **Y / N** | | | |
|  |  | **A1-A6** syphilis requiring treatment **Y / N** | | | |
|  |  | **Complicated STI:** *Was patient diagnosed with:* Epididymitis **Y / N** Pelvic inflammatory disease (PID) **Y / N** | | | |
|  |  | **Newly diagnosed HIV**: (e.g. E1A, E2A, E3A1) **Y / N** | | | |
|  | | **Patient already known to be HIV positive** (e.g. E1B, E2B) **Y / N** | | | |
| Treatment as a contact *on this date*: | | **B4** epidemiological treatment of suspected gonorrhoea **Y / N** | | | |
|  |  | **C4E** epidemiological treatment of suspected Chlamydia **Y / N** | | | |
|  |  | **C4I**  epidemiological treatment of suspected NSGI **Y / N** | | | |
|  |  | **C7B**  epidemiological treatment of trichomoniasis **Y / N** | | | |
|  |  | **A9** epidemiological treatment of suspected syphilis **Y / N** | | | |
| Treatment  /care | | **Medication Y / N** Name of drug(s): Duration of Rx (treatment):  ______________________ ________________________  ­­­­­ _____________________­­_ ________________________  **Other treatments:**  Cryotherapy **Y / N** Other *(please specify):* | | | |
| Referral to other services | | **Tick all that apply:** GP 🞏 GUM 🞏 Gynaecology 🞏  Dermatology 🞏 Termination services 🞏 HIV services 🞏 Hepatitis services 🞏  Specialist contraceptive services / family planning 🞏  Other service *(please specify):* | | | |
| **If diagnosed with Gonorrhoea / Chlamydia** (leave blank for patients without GC/CT) | | | | | |
| At least one partner reported **tested Y / N** | | | | | |
| At least one partner reported **treated Y / N** | | | | | |
